# Average Volume-assured Pressure Support as Rescue Therapy after CPAP Failure in Pediatric Obstructive Sleep Apnea: A Retrospective Case Series Study

**DOI:** 10.2174/18743064-v17-e230418-2022-18

**Published:** 2023-05-15

**Authors:** Victor T. Peng, Nauras Hwig, Anayansi Lasso-Pirot, Amal Isaiah, Montserrat Diaz-Abad

**Affiliations:** 1 Sleep Disorders Center, University of Maryland Medical Center, Baltimore, Maryland, MD 21201, USA; 2 Department of Pediatrics, Division of Pediatric Pulmonology, University of Maryland School of Medicine, Baltimore, Maryland, MD 21201, USA; 3 Department of Otorhinolaryngology-Head and Neck Surgery, University of Maryland School of Medicine, Baltimore, Maryland, MD 21201, USA; 4 Department of Diagnostic Radiology and Nuclear Medicine, University of Maryland School of Medicine, Baltimore, Maryland, MD 21201, USA; 5 Department of Medicine, Division of Pulmonary and Critical Care Medicine, University of Maryland School of Medicine, Baltimore, Maryland, MD 21201, USA

**Keywords:** Obstructive sleep apnea, Continuous positive airway pressure, Treatment, Pediatric, Noninvasive ventilation, AVAPS, Polysomnography

## Abstract

**Background:**

Continuous positive airway pressure (CPAP) is frequently prescribed for patients with residual obstructive sleep apnea (OSA) following adenotonsillectomy.

**Objectives:**

The goal was to examine the efficacy of noninvasive ventilation with average volume-assured pressure support (AVAPS) as a potential option for children with failed CPAP titration.

**Methods:**

In a single-center retrospective study, we included children aged 1-17 years, with polysomnographically confirmed OSA who underwent AVAPS titration following failed CPAP titration. In addition to describing the clinical characteristics of the included patients, we compared polysomnographic parameters before and after AVAPS.

**Results:**

Nine patients met the inclusion criteria; out of them, 8 (89%) were males with an age range of 6.7 ± 3.9 years and a body mass index percentile of 81.0 ± 28.9. Reasons for failed CPAP titration were: 3 (33%) patients due to inability to control apnea-hypopnea index (AHI), 3 (33%) patients due to sleep-related hypoventilation, 2 (22%) patients due to treatment-emergent central sleep apnea, and 1 (11%) patient due to intolerance to CPAP. AVAPS resulted in a greater reduction in AHI than CPAP (reduction following CPAP = 24.6 ± 29.3, reduction following AVAPS = 42.5 ± 37.6, p = 0.008). All patients had resolution of the problems which caused CPAP failure.

**Conclusion:**

In this case a series of children with OSA and with failed CPAP titration, AVAPS resulted in a greater reduction in AHI compared with CPAP as well as resolution of the problems which caused CPAP failure.

## 1 INTRODUCTION

Obstructive sleep apnea (OSA) is characterized by recurrent partial or complete upper airway obstruction during sleep associated with increased respiratory effort, sleep fragmentation, and/or gas exchange abnormalities [1]. Pediatric OSA has an estimated prevalence of 1.2% to 5.7% [2-4]. Untreated OSA is associated with behavioral and neurocognitive deficits, including poor school performance, learning impairments, hyperactivity, impulsivity, cardiovascular morbidity, reduced quality of life, and greater healthcare utilization [5-9].

Adenotonsillectomy is the first-line treatment in otherwise healthy children with OSA and adenotonsillar hypertrophy [2, 7, 10]. However, the prevalence of persistent OSA following adenotonsillectomy in children is as high as 25-40% [1,11]. Continuous positive airway pressure (CPAP) therapy is recommended for the management of residual OSA following adenotonsillectomy [2,12]. However, CPAP titration may fail due to multiple reasons, including persistent OSA despite CPAP, persistent hypoventilation despite CPAP, treatment-emergent central sleep apnea, and patient intolerance to CPAP therapy [13-17]. Bilevel PAP may be used when CPAP fails or is not tolerated [16].

Average volume-assured pressure support (AVAPS) is an automatically titrating mode of noninvasive ventilation, which provides a target tidal volume by adjustment of inspiratory positive airway pressure (IPAP) within a set range [13-15]. More recently, AVAPS has been used as a noninvasive ventilation strategy for the treatment of hypoventilation disorders such as chronic obstructive pulmonary disease, obesity, hypoventilation syndrome, and chronic respiratory failure [13, 18]. A study of 45 adults with OSA, who had failed CPAP titration, found AVAPS to be effective in reducing the apnea-hypopnea index (AHI, events/hour) and improving sleep architecture [15]. A randomized controlled trial of 25 adults with hypoventilation syndromes and comorbid OSA showed that intelligent volume-assured pressure support had similar efficacy in controlling sleep-disordered breathing [19].

There are limited data examining the use of AVAPS titration in children with OSA in whom CPAP titration is unsuccessful. In one case report, AVAPS was shown to be an effective therapy in a pediatric patient with severe OSA with failed CPAP titration, who was able to avoid a tracheostomy for extremely severe OSA [14]. We aimed to examine the efficacy of AVAPS titration for the treatment of OSA in pediatric patients after failure of in-laboratory CPAP titration by comparing changes in polysomnographic parameters following CPAP titration with changes following AVAPS titration.

## 2 MATERIALS AND METHODS

### 2.1 Patient Selection

We performed a retrospective review of all patients in our institution who underwent an AVAPS in-laboratory titration study between January 2017 and August 2021. The University of Maryland Institutional Review Board approved this study using previously collected data, HP-00089938. Records were reviewed for patients who met the following criteria: 1) age 1-17 years old; 2) diagnosis of OSA on a full-night in-laboratory polysomnogram (PSG); 3) previous failed in-laboratory CPAP titration study; and 4) had a follow-up in-laboratory AVAPS titration study.

### 2.2 Data Collection

Medical records were reviewed for information on age, sex, race, ethnicity, body mass index (BMI), birth complications including the history of premature birth and neonatal intensive care unit admission, comorbid medical conditions, public or private medical insurance, and prior treatment of OSA.

### 2.3 Polysomnography

In-laboratory PSGs were attended by a registered licensed polysomnographic technologist and performed using Healthdyne ALICE 4 computerized polysomnographic system (Philips Respironics, Murrysville, PA, USA) using standard technique [20]. The montage included electroencephalography, electrooculography, submentalis electromyography, lower extremity electromyography, single-lead electrocardiography, air-flow measured by the nasal pressure transducer and oronasal thermistor, thoracic and abdominal respiratory effort measured by respiratory inductance plethysmography, continuous pulse oximetry, snore microphone, and body position.

Sleep and respiratory event scoring were performed according to the standard criteria outlined in the American Academy of Sleep Medicine Manual for the Scoring of Sleep and Associated Events [20]. Respiratory events had to last ≥ 2 breaths, apneas were scored if there was a decrease in the airflow by ≥ 90% of the pre-event baseline. Hypopneas were scored if there was a decrease in the airflow by ≥ 30% of the pre-event baseline, associated with either a ≥ 3% oxygen desaturation or arousal. Obstructive apneas were scored if there was continued or increased respiratory effort throughout the entire period of absent flow. Central apneas were scored if there was an absent respiratory effort throughout the entire period of absent flow, and if at least one of the following criteria were met: (1) the event lasted ≥ 20 seconds, (2) the event was associated with ≥ 3% oxygen desaturation or arousal. Mixed apneas were scored if the respiratory effort was absent during one portion of the event with the presence of respiratory effort in another portion of the event. Oxygen desaturation indices consisted of total sleep time spent with ≤ 90% oxygen saturation during sleep (T ≤ 90%), lowest event-related oxygen desaturation, and mean oxygen saturation during the study. Duration of sleep stages as well as measures of sleep, including total sleep time, sleep efficiency, sleep onset latency, sleep stages N1, N2, N3, R, and arousal index were also recorded according to standard definitions [20]. OSA was diagnosed according to standard criteria: one or more obstructive apneas, mixed apneas, or hypopneas per hour of sleep or a pattern of obstructive hypoventilation in association with either snoring, flattening of the inspiratory nasal pressure waveform, or paradoxical thoracoabdominal motion [20].

### 2.4 CPAP Titration Study

CPAP titration was performed according to standard guidelines [20] using the Omni Lab Advanced Plus System One device (Philips Respironics, Murrysville, PA, USA). Failure of CPAP titration was determined by any of the following situations: 1) inability to reduce AHI to < 10; 2) persistent hypoventilation [defined by time with end-tidal CO_2_ level (ETCO_2_) > 50 mm Hg (T ETCO_2_ > 50) for ≥ 25% of total sleep time (TST)]; 3) development of treatment-emergent central sleep apnea (TE-CSA); and/or 5) intolerance to CPAP therapy.

### 2.5 AVAPS Titration Study

An in-laboratory AVAPS titration study was performed with the following initial settings by our institution’s standard practice: expiratory positive airway pressure (EPAP) 4 -14 cm H_2_O, pressure support 4 - 21 cm H_2_O, maximum pressure 25 cm H_2_O, timed inspiration 1.5 seconds, AVAPS rate 2, back-up respiratory rate 12 breaths/minute, and tidal volume 8 ml/kg of ideal body weight.

### 2.6 Outcome Measures

The primary outcome measure was the effectiveness of AVAPS in reducing AHI from baseline PSG compared with that of CPAP in reducing AHI from baseline PSG. Secondary outcome measures included improvements in oxygenation (as defined by the difference in T ≤ 90% and difference in minimum oxygen saturation) and improvements in sleep architecture (as defined by increased time in stage R and N3 sleep). Treatment success was defined as 1) AHI < 10 with AVAPS titration, and/or 2) the reason for CPAP titration failure was resolved on AVAPS titration.

### 2.7 Statistics

Categorical variables were assessed by counts (or frequencies) and continuous variables were assessed by means and standard deviations. Statistical significance of changes from baseline PSG between groups (CPAP, AVAPS) was determined using one-way analysis of variance (ANOVA) with associated posthoc comparisons calculated by GraphPad. The null hypothesis was rejected at the 5% level.

## 3 RESULTS

Nine patients met the inclusion criteria including 8 (89%) males, a mean age of 6.7 ± 3.9 years, and a BMI percentile of 81.0 ± 28.9%, with 6 (67%) patients considered obese. Six (67%) patients had a history of asthma and 6 (67%) patients had prior adenotonsillectomy. The reasons for failed CPAP titration were: 3 (33%) patients due to inability to control AHI, 3 (33%) patients due to sleep-related hypoventilation, 2 (22%) patients due to treatment-emergent central sleep apnea and 1 (11%) patient due to intolerance to CPAP leading to the cessation of titration. These and additional baseline characteristics and other comorbid conditions are described in Table **1**. Parameters for each sleep study in each patient are available in Table **S1**.

A comparison of sleep parameters with diagnostic PSG, CPAP titration, and AVAPS titration is described in Table **2**. Follow-up values were significantly different from the baseline (*p* = 0.003). Post-hoc testing showed a greater decrease in AHI following AVAPS = 42.5 ± 37.6, (*P* = 0.008 than CPAP = 24.6 ± 29.3 (*P* = 0.013)).

All 9 patients showed a reduction in AHI between CPAP titration and AVAPS titration, with a mean reduction of 17.9 ± 14.5. The mean reduction in T < 90 between CPAP titration and AVAPS titration of all patients was 4.7 ± 22.4 minutes. Seven (77%) patients had a reduction in T < 90 between CPAP titration and AVAPS titration with a mean reduction of 7 patients at 11.4 ± 18.9 minutes. Post-hoc testing did not demonstrate significant differences.

With AVAPS, 6 patients (67%) achieved an AHI < 10, and all achieved an AHI < 15. Furthermore, all patients experienced the resolution of the problems which caused CPAP failure. The patients who failed CPAP titration due to inability to control AHI, achieved AHI < 10 during AVAPS titration, including 1 patient with AHI < 5. All patients who failed CPAP titration due to sleep-related hypoventilation, achieved resolution of sleep-related hypoventilation during AVAPS titration (with 2 patients achieving T ETCO2 > 50 = 0 minutes, and 1 patient achieving T ETCO2 > 50 = 23 minutes, 5.7% of TST). Both patients who failed CPAP titration due to treatment-emergent central sleep apnea during the study achieved resolution; 1 patient had a central apnea index of 22.6 during CPAP titration and a central apnea index of 1 during AVAPS titration, while 1 patient had a central apnea index of 3.6 during CPAP titration and central apnea index of 0 during AVAPS titration. One of the patients failed CPAP titration due to intolerance to pressure and tolerated the AVAPS titration.

## 4 DISCUSSION

This study reports the use of AVAPS as a treatment modality for pediatric patients with OSA, who have failed CPAP titration, this novel mode of noninvasive ventilation was successful as rescue therapy in all patients, either by achieving an AHI < 10 or by resolving the reason for CPAP titration failure. There was a significant improvement in OSA from baseline PSG between CPAP titration and AVAPS titration, with a marked reduction in AHI. No significant changes in sleep architecture were found.

Obstructive sleep apnea results from a combination of abnormal neuromuscular control and anatomic narrowing of the upper airway. Decreased ventilatory drive and neuromuscular tone during sleep facilitate upper airway collapse, leading to recurrent hypoxemia, hypercapnia, and sleep disruption [21]. For pediatric patients with OSA and adenotonsillar hypertrophy, adenotonsillectomy remains the first-line treatment in most cases [2, 7, 10], while CPAP therapy is recommended as second-line therapy in the case of refractory OSA [2]. Observational clinical studies have shown that treatment with CPAP can improve symptoms and PSG findings in up to 85% of children diagnosed with OSA [1, 22]. An attended in-laboratory titration is considered the gold standard for the initiation of CPAP therapy; however, CPAP titration can fail for multiple reasons [16]. As seen in our patient population, reasons for CPAP titration failure include the inability to control AHI, sleep-related hypoventilation, treatment-emergent central sleep apnea, or CPAP intolerance.

While some studies have indirectly reported the incidence of CPAP failure in adults with OSA as approximately 25% [23], there are no studies that examine the incidence or specific causes of CPAP titration failure in pediatric patients. Furthermore, there are few guidelines and published evidence regarding the management of CPAP titration failure in pediatric patients. One of the studies has reported the efficacy of using therapy with high-flow heated and humidified air via nasal cannula in pediatric patients with CPAP intolerance [24]. In adult patients, commonly used modalities for CPAP failure include bilevel PAP for pressure intolerance, adaptative servo-ventilation and bilevel PAP to back-up respiratory rate for treatment-emergent central sleep apnea [15]. A prospective nonblinded, non-randomized study of adult patients reported that auto-titrating bilevel PAP led to substantial improvement of AHI in patients with CPAP intolerance and inadequate control of AHI on CPAP [17]. A prospective randomized crossover clinical trial of adult patients reported adaptive servo-ventilation led to a significantly improved AHI and respiratory arousal index in patients with CPAP failure due to treatment-emergent central sleep apnea, and with a greater effect compared to traditional bilevel PAP [25].

A recent review of the current literature behind the use of AVAPS in the pediatric population described several studies that found the efficacy of AVAPS in several conditions compared with traditional bilevel PAP. These included an improved treatment of hypoventilation and improved adherence [26]. However, the authors noted that the body of evidence on the use of AVAPS in pediatric patients remains confined to a single center and case studies without control groups, and highlighted the need for prospective randomized controlled trials [26].

Our study is one of the first to evaluate the potential efficacy of AVAPS treatment in a small group of pediatric patients with OSA who failed CPAP titration. AVAPS led to a significant reduction in AHI with CPAP titration in all patients. We were also able to achieve a resolution of the reasons for the failure of CPAP titration in all patients, including the inability to control AHI, sleep-related hypoventilation, treatment-emergent central sleep apnea, and CPAP intolerance. Consistent with our findings, a case report examining AVAPS therapy in a pediatric patient with morbid obesity, severe OSA, and failed CPAP titration demonstrated successful treatment of the condition, avoiding tracheostomy [14]. Another study included a retrospective case series of 19 pediatric patients comparing AVAPS titration to conventional bilevel PAP titration in treating pediatric nocturnal hypoventilation but was not specifically focused on OSA. The authors reported significant improvement in nocturnal hypoventilation with AVAPS compared to conventional bilevel PAP, although without significant changes in sleep efficiency, AHI, respiratory arousal index, or adherence to treatment [18]. The advantage over conventional bilevel PAP was thought to be secondary to the ability of AVAPS to maintain a constant tidal volume during alterations of respiratory effort in different sleep stages, as well as due to altered lung compliance secondary to parenchymal lung disease [18].

In a similar study, a retrospective review of 45 adult patients with OSA examined the efficacy of AVAPS after the failure of CPAP titration. Reasons for CPAP titration failure included treatment-emergent central sleep apnea and inability to control AHI. The authors reported a significant improvement in the AHI and oxygen saturation between baseline PSG and AVAPS titration, while oxygen saturation was not significantly improved between PSG and CPAP titration [15].

While it is a common practice following initiation of PAP to perform repeat CPAP titration to reevaluate the therapeutic pressure [27], a recent retrospective review of 64 pediatric patients with OSA demonstrated that no significant changes in PAP settings were needed when a repeated PAP titration was performed within two years, and concluded titration within such an interval is unnecessary [27]. However, in some children, intervals greater than two years may conceivably lead to growth-related changes that would necessitate changes in PAP pressure. In such patients, AVAPS may present a potential advantage in reducing the need for frequent repeat PAP titrations to treat OSA.

Our study has several limitations. The primary drawback is the retrospective nature of the study with a small sample size of nine patients at a single medical center, thereby limiting the conclusion related to statistical tests. Furthermore, our patients were predominately male and obese and therefore may limit the generalizability of the findings to all OSA patients. In addition, our study did not compare AVAPS with conventional bilevel PAP therapy, which is often the next step after CPAP failure [16]. A prospective randomized blinded controlled clinical trial with patients receiving either conventional bilevel PAP or AVAPS would need to be conducted to draw firm conclusion regarding the effectiveness of AVAPS in CPAP failure, as well as its relative utility compared to conventional bilevel PAP which is a more commonly used modality of noninvasive ventilation. Despite these limitations, given our findings and those of prior studies, AVAPS therapy may reasonably be considered as an alternative therapeutic modality in selected pediatric patients with OSA in whom CPAP therapy is unsuccessful.

## CONCLUSION

To summarize, this study reports the use of AVAPS in pediatric patients with OSA who failed CPAP titration. AVAPS was successful as a rescue therapy in all patients, either by achieving an AHI < 10 or by resolving the reason for CPAP titration failure. Due to the harms associated with untreated OSA in this population, continued research into effective alternative therapies is indicated. Future studies with prospective, randomized controlled clinical trials to determine the efficacy of AVAPS in this population are needed.

## Figures and Tables

**Table 1:** Baseline characteristics and comorbidities.

**S.No**	**Sex**	**Age (y)**	**Race**	**Ethnicity**	**Insurance**	**BMI %ile**	**Comorbidities**	**Prior T & A**	**Reason for Failed CPAP Titration**
1	Male	8	Other	Hispanic or Latino	Public	99.9	Prematurity	No	AHI ≥ 10
2	Male	5	White	Not Hispanic or Latino	Private	12.5	None	Yes	Hypoventilation
3	Male	11	White	Not Hispanic or Latino	Public	99.9	Asthma	No	TE-CSA
4	Male	11	Black	Not Hispanic or Latino	Public	99.6	Asthma	Yes	Hypoventilation
5	Male	2	Black	Not Hispanic or Latino	Public	66.7	Croup, bronchitis, speech delay	Yes	CPAP intolerance
6	Male	4	Black	Not Hispanic or Latino	Public	99.9	Asthma, allergic rhinitis	Yes	AHI ≥ 10
7	Male	3	Black	Not Hispanic or Latino	Public	55.6	Sickle cell disease, asthma, allergic rhinitis	Yes	TE-CSA
8	Male	3	Black	Not Hispanic or Latino	Public	95.5	Kabuki syndrome, atrial septal defect, asthma	Yes	Hypoventilation
9	Female	13	Black	Not Hispanic or Latino	Public	99.9	Atrial septal defect, asthma, allergic rhinitis	No	AHI ≥ 10

**Abbreviations:** AHI: apnea-hypopnea index (events/hour); BMI: body mass index; T & A: tonsillectomy and adenoidectomy; TE-CSA: treatment-emergent central sleep apnea.

**Table 2:** Comparison between sleep parameters on diagnostic PSG, CPAP, and AVAPS.

**Parameter**	**PSG**	**CPAP**	**AVAPS**	**Difference between CPAP and PSG**	**Difference between AVAPS and PSG**	**P (overall)**	**P (PSG)**	**P (CPAP)**	**P (AVAPS)**
AHI (events/h)	49.5 ± 39.5	25.0 ± 15.2	7.1 ± 3.9	24.6 ± 29.3	42.5 ± 37.6	**0.002647**	0.0449	0.0127	0.0082
Time oxygen saturation ≤ 90% (min)	18.2 ± 33.6	9.0 ± 17.2	4.2 ± 11.4	9.3 ± 18.0	14.0 ± 37.5	0.361325	N/A	N/A	N/A
Minimum oxygen saturation (%)	70.6 ± 11.8	81.4 ± 9.7	90.1 ± 4.2	-10.9 ± 8.4	-19.6 ± 14.1	**0.000724**	0.0064	0.0043	0.0589
Total sleep time (min)	343.7 ± 71.8	322.8 ± 94.0	370.6 ± 71.1	20.9 ± 96.0	-26.7 ± 116.9	0.414188	N/A	N/A	N/A
Sleep efficiency (%)	79.3 ± 13.1	74.6 ± 20.5	82.7 ± 15.7	4.7 ± 22.9	-3.4 ± 23.7	0.559691	N/A	N/A	N/A
N3 (%)	22.6 ± 5.4	21.0 ± 6.6	19.5 ± 7.7	1.6 ± 10.1	3.1 ± 10.1	0.70275	N/A	N/A	N/A
R (%)	12.0 ± 3.8	9.6 ± 7.1	12.9 ± 8.3	2.4 ± 7.6	-0.9 ± 9.1	0.458038	N/A	N/A	N/A
Sleep latency (min)	29.6 ± 29.1	24.3 ± 19.5	42.9 ± 59.6	5.3 ± 41.1	-13.4 ± 73.9	0.651948	N/A	N/A	N/A
Arousal index (arousals/h)	38.6 ± 36.1	12.1 ± 15.4	1.3 ± 1.4	26.5 ± 27.4	37.3 ± 36.0	**0.004518**	0.0256	0.019	0.0869

## Data Availability

The data and supportive information is available within the article.

## LIST OF ABBREVIATIONS

OSA: = Obstructive sleep apnea
CPAP: = Continuous positive airway pressure
AVAPS: = Average volume-assured pressure support
EPAP: = Expiratory positive airway pressure
AHI: = Apnea-hypopnea index
PSG: = Polysomnogram
BMI: Body mass index
T ≤ 90%: = Total sleep time spent with ≤ 90% oxygen saturation sleep
T ETCO_2_ > 50: = Time with ETCO_2_ > 50 mm Hg
TST: = Total sleep time
TE-CSA: = Treatment-emergent central sleep apnea
