# Supplementary material for: Average Volume-assured Pressure Support as Rescue Therapy after CPAP Failure in Pediatric Obstructive Sleep Apnea: A Retrospective Case Series Study
Source: Open Respir Med J. 2023 May 15;17:e187430642303080. doi: 10.2174/18743064-v17-e230418-2022-18 (PMC10351336; doi:10.2174/18743064-v17-e230418-2022-18)
Supplement: Supplementary file 1 [file TORMJ-17-E187430642303080_SD1.pdf]

1874-3064/23 *Send Orders for Reprints to reprints@benthamscience.net*

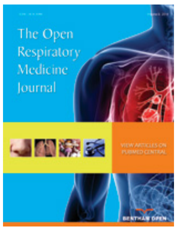

The Open Respiratory Medicine Journal

Content list available at: https://openrespiratorymedicinejournal.com

Supplementary Material

CrossMark

Average Volume-assured Pressure Support as Rescue Therapy after CPAP Failure in Pediatric Obstructive Sleep Apnea: A Retrospective Case Series Study

Victor T. Peng[1], Nauras Hwig[1], Anayansi Lasso-Pirot[2], Amal Isaiah[3,4] and Montserrat Diaz-Abad[5,*]

[1]*Sleep Disorders Center, University of Maryland Medical Center, Baltimore, Maryland, MD 21201, USA*
[2]*Department of Pediatrics, Division of Pediatric Pulmonology, University of Maryland School of Medicine, Baltimore, Maryland, MD 21201, USA*
[3]*Department of Otorhinolaryngology-Head and Neck Surgery, University of Maryland School of Medicine, Baltimore, Maryland, MD 21201, USA*
[4]*Department of Diagnostic Radiology and Nuclear Medicine, University of Maryland School of Medicine, Baltimore, Maryland, MD 21201, USA*
[5]*Department of Medicine, Division of Pulmonary and Critical Care Medicine, University of Maryland School of Medicine, Baltimore, Maryland, MD 21201, USA*

Supplemental Table 1. Sleep study parameters in each individual patient.

Patient #	AHI (events/h)			Time Oxygen Saturation ≤ 90% (min)			Minimum Oxygen Saturation (%)			Total Sleep Time (min)			Sleep Efficiency (%)			N3 (%)			R (%)			Sleep Latency (min)			Arousal Index (arousals/h)			Reason For Failed CPAP Titration
Study Type	P	C	A	P	C	A	P	C	A	P	C	A	P	C	A	P	C	A	P	C	A	P	C	A	P	C	A	
1	138.2	57.5	9.7	111.7	55.9	0	59	72	93	323.5	358	429	78.5	82.3	97.1	15.4	29.2	16.4	5	12.8	20	0.5	3	3	122.4	50.6	0.3	AHI ≥ 10
2	90	24.7	12.3	11.5	0.5	0	75	87	95	395.5	384.5	380	91.6	86	86.4	24	20.5	10.3	12.4	7.6	11	13	46	21	74.5	3.7	2.5	Hypoventilation
3	40.7	27.7	10.7	1.5	0.6	1.2	83	88	83	405.5	162.5	186	91.1	33.9	43.1	35	10	16.1	17.8	2.2	0	0	49.5	196	29.1	25.1	1	TE-CSA
4	18.6	3.9	1.6	3.3	0	0	80	96	95	399.5	352	405	92.3	83	92	18.8	12.5	22.4	17.3	22.1	31.8	25.5	24	6.5	16.5	0.3	0.3	Hypoventilation
5	64.5	27.5	4.8	7.8	6.4	0	60	70	94	154.5	181	401.5	48.7	46.3	89.8	17.7	15	22	8.8	0	8.1	29.5	55	25	42.3	0.7	1.5	CPAP intolerance
6	30.4	38.9	2.5	7	15.3	0	64	65	89	365.5	335	389.5	81.6	84.4	78.5	24.7	25.6	27	11.2	8.6	11.3	32.5	12	90	19.9	4.5	0.8	AHI ≥ 10
7	8.8	9.1	3.2	0	0	36.5	91	88	85	346	456.5	428.5	71	96.7	98.3	21.4	23.3	35.8	11.5	20.2	10.3	20	9	6	1.2	4.5	0.1	TE-CSA
8	38.9	16.1	11.5	20.1	0.6	0.5	69	87	87	349	406	328.5	73.5	94	77.5	21.1	28.6	12.4	13.5	7.2	12.4	102.5	2	12.2	35.4	10.5	4.6	Hypoventilation
9	15.7	19.3	7.4	1.2	1.5	0	54	80	90	354.5	270	387.5	85.6	64.6	82	24.9	24.3	11.5	10.4	5.6	11.5	42.5	18	26.6	5.8	8.9	0.6	AHI ≥ 10

Abbreviations: A: AVAPS titration; AHI: apnea-hypopnea index; P: baseline polysomnogram; C: CPAP titration; TE-CSA: treatment-emergent central sleep apnea.

© 2023 Peng *et al*.

This is an open access article distributed under the terms of the Creative Commons Attribution 4.0 International Public License (CC-BY 4.0), a copy of which is available at: https://creativecommons.org/licenses/by/4.0/legalcode. This license permits unrestricted use, distribution, and reproduction in any medium, provided the original author and source are credited.

DOI: 10.2174/18743064-v17-e230418-2022-18, 2023, *17*, e187430642303080
